# Intergenerational longitudinal associations between parental reading/musical traits, infants’ auditory processing, and later phonological awareness skills

**DOI:** 10.3389/fnins.2023.1201997

**Published:** 2023-07-19

**Authors:** Chiara Cantiani, Chiara Dondena, Massimo Molteni, Valentina Riva, Maria Luisa Lorusso

**Affiliations:** Child Psychopathology Unit, Scientific Institute, IRCCS Eugenio Medea, Lecco, Italy

**Keywords:** reading, musical aptitude, EEG/ERP, auditory processing, phonological awareness, intergenerational transmission

## Abstract

The intergenerational transmission of language/reading skills has been demonstrated by evidence reporting that parental literacy abilities contribute to the prediction of their offspring’s language and reading skills. According to the “Intergenerational Multiple Deficit Model,” literacy abilities of both parents are viewed as indicators of offspring’s liability for literacy difficulties, since parents provide offspring with genetic and environmental endowment. Recently, studies focusing on the heritability of musical traits reached similar conclusions. The “Musical Abilities, Pleiotropy, Language, and Environment (MAPLE)” framework proposed that language/reading and musical traits share a common genetic architecture, and such shared components have an influence on the heritable neural underpinnings of basic-level skills underlying musical and language traits. Here, we investigate the intergenerational transmission of parental musical and language-related (reading) abilities on their offspring’s neural response to a basic auditory stimulation (neural intermediate phenotype) and later phonological awareness skills, including in this complex association pattern the mediating effect of home environment. One-hundred and seventy-six families were involved in this study. Through self-report questionnaires we assessed parental reading abilities and musicality, as well as home literacy and musical environment. Offspring were involved in a longitudinal study: auditory processing was measured at 6  months of age by means of a Rapid Auditory Processing electrophysiological paradigm, and phonological awareness was assessed behaviorally at 5  years of age. Results reveal significant correlations between parents’ reading skills and musical traits. Intergenerational associations were investigated through mediation analyses using structural equation modeling. For reading traits, the results revealed that paternal reading was indirectly associated with children’s phonological awareness skills via their electrophysiological MisMatch Response at 6  months, while maternal reading was directly associated with children’s phonological awareness. For musical traits, we found again that paternal musicality, rather than maternal characteristics, was associated with children’s phonological phenotypes: in this case, the association was mediated by musical environment. These results provide some insight about the intergenerational pathways linking parental reading and musical traits, neural underpinnings of infants’ auditory processing and later phonological awareness skills. Besides shedding light on possible intergenerational transmission mechanisms, this study may open up new perspectives for early intervention based on environmental enrichment.

## 1. Introduction

Language and music are complex, universal human abilities. Although they both sound different from one culture to another, they share common ground at the acoustic and structural level and they seem to develop following similar milestones, e.g., 9-month-old infants can easily discriminate native speech sounds as well as native musical sounds but not non-native speech and musical sounds; furthermore, toddlers start showing syntactic competence for their native language as well as for their culture’s musical system around 2–3 years of age (Hannon and Trainor, 2007; Brandt et al., 2012). Moreover, several theories have outlined shared underlying mechanisms for musical and language processing (Goswami, 2011, 2018; Patel, 2011, 2012; Tierney and Kraus, 2014). The recently developed *processing rhythm in speech and music* (PRISM) framework (Fiveash et al., 2021) highlighted three common elements in these theories, emphasizing the shared role of fine-grained auditory processing, synchronization/entrainment of neural oscillations, and sensorimotor coupling. In addition, a majority of studies show strong associations between musical abilities and different aspects of typical and atypical language competence across the lifespan (Corriveau and Goswami, 2009; Zuk et al., 2013; Colling et al., 2017; Ladányi et al., 2020). In light of such associations, it is now often reported that modulating musical ability can result in a beneficial effect on language skills (e.g., Lorenzo et al., 2014; Cancer and Antonietti, 2022). This modulation can be accomplished thanks to musical-based training, which provides guided and greater exposure to rhythmic musical activities (Moreno et al., 2009; Bhide et al., 2013; Flaugnacco et al., 2015; Dondena et al., 2021).

While much of this research has focused on the behavioral overlap between music and speech and on the shared underlying processes, much less research exists on the genetic and environmental contributions leading to the development of neural systems for music and language. To date, the genetic architectures of language-related and musical traits have been primarily studied separately. As recently highlighted by a meta-analysis of existing twin studies on language, reading, and related traits (Andreola et al., 2021), many of these traits are moderately heritable (with heritability estimates of 30 to 80% depending on the trait). A recent large-scale genome-wide association study (GWAS) of individual differences in reading- and language-related phenotypes in sample sizes of up to ∼34,000 participants demonstrated significant heritability based on Single Nucleotide Polymorphisms (SNPs) for all traits (Eising et al., 2022). Studies including children at familial risk for dyslexia have shown an important intergenerational transmission of language and language-related traits, estimating that if a child has a parent with dyslexia, their own probability of having dyslexia is on average 45% (see Snowling and Melby-Lervåg, 2016 for a meta-analysis). Further studies have shown that parental reading explains a significant percentage of children’s reading fluency (van Bergen et al., 2017), with both self-reported and directly assessed parental reading ability being significant predictors of children’s reading fluency and accuracy (Khanolainen et al., 2022).

Other studies have focused on understanding whether and to what extent musical skills are inherited, but the picture is less clear. A few studies initially focused on the role of parents’ own involvement and aptitude toward musical activities on the offspring’s musical development and competence (Davidson et al., 1996; McPherson, 2009). Similar to studies conducted on reading and language-related traits, twin and family-based studies have shown that musical phenotypes have a significant genetic component, with heritability estimates of 50 to 80% depending on the trait (Drayna et al., 2001; Seesjärvi et al., 2016). Specifically, pitch discrimination ability seems to be primarily determined by genetic factors, whereas the perception of metric structures seems to be more mediated by environmental factors (Seesjärvi et al., 2016). More recently, the heterogeneity and complexity of this phenotype has been further investigated, and genetic variants have been found to be associated with different components of musicality (Niarchou et al., 2022; Gustavson et al., 2023). A very recent GWAS (Niarchou et al., 2022) has reported the polygenic nature of the ability to synchronize to a beat; its heritability is most evident in those brain regions that are involved in rhythm perception, i.e., motor and auditory regions.

Recently, the *Musical Abilities, Pleiotropy, Language, and Environment (MAPLE) framework* has been proposed (Nayak et al., 2022). According to this framework, the behavioral associations reported in the literature between musical and language-related abilities could be partly driven by a shared genetic architecture (i.e., genetic pleiotropy, and more specifically polygenic pleiotropy, since complex trait phenotypes, such as both music and language, are typically polygenic). This shared genetic component is proposed to exert an influence on the development and functioning of the neural networks responsible for basic-level skills underlying musical and language traits, such as auditory processing or sensorimotor coordination. The neural underpinnings of these low-level skills are seen as potential neural endophenotypes, i.e., intermediate biological phenotypes that are heritable and, being more elementary and straightforward measures of functioning than the clinical phenotype, are functionally involved in the relationship between a genotype and a phenotype (Gottesman and Gould, 2003; Flint and Munafò, 2007). In the MAPLE framework, auditory processing and sensorimotor coordination are suggested to mediate the relationship between the genetic architecture and the music/language phenotypes. Interestingly, the genetic influences on musicality and language are also thought to act on some key environmental factors, such as home musical and language environments. In other words, the genetic predispositions of parents are thought to influence the home environments during early development, for example when musically talented parents choose to provide an enriched musical environment for their children or when skilled reading parents are more likely to spend time reading with their children, thus providing an enriched literacy environment. In this sense, this framework is consistent with the “intergenerational multiple deficit model” (van Bergen et al., 2014b), suggesting that the literacy abilities of both parents are viewed as indicators of their offspring’s liability for literacy difficulties, since parents provide offspring with genetic and environmental endowment.

Here we focus on low-level processing of the acoustic signal as a necessary element underlying both language/reading and music and as a potential endophenotype for language-related and musical traits. A large literature has emphasized the role of low-level auditory processing, including Rapid Auditory Processing (RAP), in terms of processing brief and rapidly occurring successive auditory cues, in language and reading development (e.g., Tallal and Gaab, 2006; Hämäläinen et al., 2013; Goswami, 2022). Specifically, the accurate processing of these specific features in the auditory input has been hypothesized to be directly implicated in the building of accurate phonological representations (Tallal and Gaab, 2006). Conversely, poorly organized or poorly refined phonological representations are suggested to result in difficulties in manipulating individual phonemes in spoken words, a skill that is defined as phonological awareness, and that is strictly related to reading acquisition (Tallal and Gaab, 2006). Therefore, RAP deficits affect speech perception, leading to phonological awareness deficits and subsequent reading problems, as additionally suggested by evidence of a functional relationship between neuronal networks for RAP and phonological processing/awareness within the pre-reading brain (Pugh et al., 2013; Raschle et al., 2014). Longitudinal studies have shown that early auditory processing is (a) impaired in infants with a family history of language and learning impairments (Van Leeuwen et al., 2006; van Herten et al., 2008; Leppänen et al., 2010; Choudhury and Benasich, 2011; Van Zuijen et al., 2012; Plakas et al., 2013; Cantiani et al., 2016, 2019; Lohvansuu et al., 2018; Thiede et al., 2019), and (b) highly predictive of later language and reading development both in typically developing infants and in infants at familial risk (Guttorm et al., 2005; Leppänen et al., 2010; Choudhury and Benasich, 2011; Van Zuijen et al., 2013; Cantiani et al., 2016, 2019; Piazza et al., 2016; Lohvansuu et al., 2018; Kalashnikova et al., 2019).

Previous studies have additionally shown that such low-level auditory processing skills are highly heritable (Dionne et al., 2013; Brewer et al., 2016), with heritability estimates ranging from 31 to 74% (Brewer et al., 2016), satisfying most of the criteria to be considered a solid endophenotype of developmental dyslexia (Mascheretti et al., 2018). An assumption of the intermediate phenotype approach is that the genetic susceptibility of the dysfunction of a particular neural system is likely to be relatively less complex, with a simpler causal and etiological structure, than that of the illness phenotype overall. To better understand the link between genetic mechanisms and complex phenotypes such as language or reading, researchers have recently used neuroimaging techniques and event-related potentials (ERPs). For example, in a longitudinal design, Riva et al. (2017) successfully used an electrophysiological paradigm tapping RAP at 6 months of age as a potential neural endophenotype, mediating the relationship between a common variant in the gene CNTNAP2 and language phenotype (i.e., expressive vocabulary at 20 months). The same electrophysiological paradigm has been used in the present study, allowing us to analyze two aspects of low-level auditory processing skills, reflected in two well-defined neural signatures: (1) basic auditory detection and registration (i.e., detection of and orienting to the presented sound), as reflected in the positively-displaced P1 peak obligatorily elicited by stimulus occurrence (Čeponiene et al., 2002, 2005, 2008; Ortiz-Mantilla et al., 2012), and (2) auditory discrimination, as reflected in the MisMatch Response (MMR), defined as the large positivity elicited by change detection (Kushnerenko et al., 2002). The multi-feature nature of the paradigm allowed us to investigate neural discrimination to two different acoustic features (i.e., changes in fundamental frequency and variation in sound duration). In previous studies from our group (e.g., Cantiani et al., 2016), we hypothesized that changes in fundamental frequency would be closely related to fine-grained acoustic analysis, and changes in sound duration would be more related to slowly-varying envelope changes (i.e., to the analyses of the rhythmic timing).

Focusing on this electrophysiological paradigm, in the present study, we analyzed data from a broad longitudinal Italian sample (Cantiani et al., 2016; Riva et al., 2019) in order to investigate some predictions from the MAPLE framework (Nayak et al., 2022). We employed an intergenerational longitudinal design. First, we collected data from both parents, concerning their reading abilities and their attitudes toward melodic and rhythmic properties of music. Second, we collected longitudinal data from their children: at 6 months of age we collected infants’ electrophysiological responses to a low-level auditory stimulation including changes in frequency and duration (basic auditory detection, as reflected in the P1 peak, and auditory discrimination, as reflected in the MMR). Four and half years later, we collected children’s phonological awareness skills, as a measure of their ability to recognize and manipulate the spoken parts of words (i.e., syllables and phonemes). Finally, we collected information about the environment children were exposed to; specifically, we collected data about the musical activities children were exposed to with their mothers and fathers in the course of the first year of life, and the overall literacy environment children were exposed to in the first three years of life. Through this large and diversified dataset, we empirically explored the following research questions:

1. *Are reading skills and musical aptitudes associated?* Associations between reading skills and melodic vs. rhythmic musical aptitudes were investigated in the sample of adults included in the study as parents.
2. *Are parental reading skills/musical aptitudes associated with their offspring’s auditory processing and phonological awareness?* In an intergenerational longitudinal design, we explored the associations between parental reading skills/musical aptitudes and their children’s skills. More specifically, the neural correlates of low-level auditory processing (P1 and MMR) were included as a possible intermediate phenotype common to the language and music system, and phonological awareness skills at 5 years of age as a reading-related trait.
3. *Do parental reading skills and their musical aptitudes influence the environment (respectively the literacy and musical environment) provided to children? How does this environment influence children’s auditory processing and phonological awareness?* In the intergenerational longitudinal design, we further explored how parental reading skills/musical aptitudes influence the environment provided to children and what role such an environment plays in children’s development.

Since both reading and musical aptitudes are heritable traits (e.g., Andreola et al., 2021; Eising et al., 2022; Niarchou et al., 2022) we followed the “intergenerational multiple deficit model” for dyslexia (van Bergen et al., 2014b) and treated these parental phenotypes as a proxy for their genotypes, expecting associations with their children’s endophenotypes and phenotypes. When available, we investigated maternal and paternal influences separately, in order to disentangle the parent-specific intergenerational pathways, since some forms of parent-of-origin effects have been reported for some complex traits, including language-related development (Nudel et al., 2022). Since individual differences in parental reading and musical skills cannot be attributed to genetic influences alone, we additionally expected the home environment to be implicated in these complex relationships. The role of home literacy environment in children’s language and reading development is well documented (e.g., Burgess et al., 2002; Park, 2008; Dong et al., 2020; Niklas et al., 2020), and similarly the effect of early informal musical activities at home on language development has been recently reported (Williams et al., 2015; Politimou et al., 2019; Schaal et al., 2020; Papadimitriou et al., 2021; Franco et al., 2022).

Based on this literature, we specifically expected (1) reading skills and musical aptitudes to be associated (Nayak et al., 2022); based on previous studies, we might expect timing and melodic skills to be independently associated with language-related skills (Politimou et al., 2019); (2) parental reading skills/musical aptitudes to be associated with their offspring’s phonological awareness skills both directly (van Bergen et al., 2017; Khanolainen et al., 2022) and indirectly (mediated by the neural correlates of low-level auditory processing skills; Van Zuijen et al., 2012; Plakas et al., 2013; Thiede et al., 2019); (3) parental skills to influence the literacy and musical environment their children are exposed to, that in turn is expected to exert a role in children’s development (e.g., Dong et al., 2020; Niklas et al., 2020; Papadimitriou et al., 2021; Franco et al., 2022). The environmental influence on children’s development could be directly driven by parental phenotypes, or associated with parental genotypes, creating what is called a “passive gene–environment correlation” (van Bergen et al., 2014b).

## 2. Materials and methods

### 2.1. Participants

Infants of 6 months of age and their parents were recruited to take part in a longitudinal study (Cantiani et al., 2016, 2019; Riva et al., 2017) which follows children up until primary school. The study was approved by the Medea Institute’s Scientific and Ethical Committees and all parents gave their written consent prior to testing.

Infants’ electrophysiological data were collected at approximately 6 months of age (*M* = 6.6 months, *SD* = 0.5) through a Rapid Auditory Processing task (see paragraph 2.4) and concurrent cognitive development was assessed using the Bayley Scales of Infant and Toddler Development (Bayley, 2006). Data concerning phonological awareness were successively collected during a follow-up session scheduled at approximately 5 years of age (see paragraph 2.5). Furthermore, parents’ reading abilities were assessed using questionnaires and the commonly used standardized tests. Parents’ musical skills (both melodic and rhythmic skills) were assessed using questionnaires. Lastly, data concerning literacy and musical environment were collected (see paragraphs 2.2 and 2.3). All parental measures were collected between infants’ 6- and 12-month-old experimental sessions, with the exception of the literacy environment data which were collected at children’s 3-year-old session.

For the purpose of this study, we considered four elements: (1) infants’ EEG/ERP data at 6 months, (2) children’s phonological awareness data at 5 years, (3) parents’ reading and musical assessment, (4) information about literacy and musical environment. Not every measure was available for all families, e.g., due to scheduling problems for parents’ reading assessment, rejection of EEG/ERP data, or because children had not yet reached the 5-year-old session; furthermore, the questionnaire assessing musical skills and environment was added to the research protocol only for later participants. Therefore, families were included in the sample when at least two out of these measures were available. Other inclusion criteria were (1) both parents were Italian native speakers, (2) infants’ gestational age was ≥35 weeks, (3) infants’ Bayley Cognitive Scale Score at 6 months of age was ≥7.

The final sample consisted of 176 families in which the mother, the father and one child (87 males) took part in the study. In 42 of these families, a parent (*n* = 2) or an older sibling (*n* = 40) had a certified diagnosis of Developmental Language Disorder (*n* = 21), Developmental Dyslexia (*n* = 11) or both (*n* = 10). Table 1 shows descriptive statistics for the whole sample.

**Table 1:** Descriptive statistics for socio-demographic and anamnestic data.

	Mean (*SD*)	Min	Max
PARENTS			
Maternal education level^a^	57.10 (16.39)	20	80
Paternal education level^a^	47.95 (17.22)	20	80
Socio-economic status^b^	64.08 (16.94)	30	90
Maternal age at delivery (years)	33.86 (4.57)	20	48
Paternal age at delivery (years)	36.85 (5.22)	26	52
INFANTS			
Gestational age (weeks)	39.40 (1.53)	35	42
Bayley Cognitive scaled score (6 months)	11.93 (1.64)	7	16

a Eight-point ordinal scale, ranging from 10 to 80, created *ad hoc* and based on the Italian school system (Hollingshead, 1975). Scores ranged between 10, corresponding to the fifth grade of elementary school (with 50 corresponding to high-school diploma) and 80, corresponding to post-doctoral degree. Information about the level of school completed by each parent was collected using an *ad-hoc* created questionnaire.

b Nine-point scale, whereby a score ranging from 10 to 90 was assigned to each parental job and the higher of the two scores was used when both parents were employed (Hollingshead, 1975). Scores ranged between 10, corresponding to unskilled workers (with 50 corresponding to sales workers) and 90, corresponding to major professionals. Information about each parent’s occupational title was collected using an *ad-hoc* created questionnaire.

### 2.2. Parental assessment: reading and literacy

#### 2.2.1. Reading skills

A self-report measure of parents’ own reading abilities was collected using the Adult Dyslexia CheckList (ADCL; Vinegrad, 1994) questionnaire. It is a widespread screening tool for adults with dyslexia (Pinnelli and Cursi, 2010), composed of twenty questions with Yes/No answers; each positive answer indicates difficulty. The total number of “Yes” answers was entered in the analysis. This measure was available for 172 mothers and 172 fathers.

In addition to self-report measures, a subgroup of parents was also evaluated directly for their reading accuracy and speed. Standardized tests assessing text reading (Judica and De Luca, 2005) and single-word (4 lists of 28 words each) and pseudo-word (3 lists of 16 non-words each) reading (Sartori et al., 1995) were administered by a psychologist during individual sessions with each parent. Normative z-scores for accuracy and speed were calculated for each task and considered for the analysis. These measures were available for 111 mothers and 94 fathers.

For the purpose of the present manuscript, we used the measures of direct reading assessment (available in a subsample of parents) to validate self-reported measures, thus preliminarily exploring the relationship between task-based reading ability and self-reported measures.

#### 2.2.2. Literacy environment

The Home Literacy Environment Questionnaire (HLEQ; Umek et al., 2005) was collected when children were three years old. The Italian adaptation of the HLEQ is composed of 33 statements that investigate the quality and quantity of stimulation of literacy skills children are exposed to in their family environment, e.g., how correctly parents talk to their children in terms of grammar, syntax and vocabulary, how much they encourage them to speak correctly, how often they engage in different interactive literacy activities such as reading books or visiting libraries/theaters. Parents have to score the frequency of each behavior on a 3-point scale. The sum of raw scores was entered in the analysis. Each family filled the questionnaire once, therefore only an overall home literacy score was available, instead of separate scores for mothers and fathers. This measure was available for 141 families.

### 2.3. Parental assessment: musical traits

#### 2.3.1. Musical skills: self-report

To estimate their musical sense and skills, parents were asked to fill out the Brief Music Experience Questionnaire (BMEQ; Werner et al., 2006), a 53-item self-report measure of individual differences in people’s experience of music. Each item is scored on a 5-point Likert scale, and the scoring produces six scales: Commitment to music, Innovative musical aptitude, Social uplift, Affective reactions, Positive psychotropic effects and Reactive musical behavior. For the purpose of this study, only those scales assessing musical skills were considered: Innovative musical aptitude, concerning sense of melody and singing/playing abilities, and Reactive musical behavior, concerning the sense of rhythm and synchronization. The remaining scales, which assess emotions and feelings toward music, were not considered. The mean score for each scale (ranging from 1 to 5) was used for the analysis. These measures were available for 87 mothers and 68 fathers.

#### 2.3.2. Musical environment

Mothers and fathers separately completed a short *ad-hoc* questionnaire concerning musical environment, measured in how much they engaged in joint musical activities with their children during their first year of life. For example, each parent had to estimate how much time (quantified in average minutes per day) they spent listening to music with their child, singing to their child, and lulling their child rhythmically. A total score comprehensive of all musical activities for each parent was used for the analysis (i.e., the sum of the average minutes per day spent in each activity). This measure was available for 71 mothers and 51 fathers.

### 2.4. Electrophysiological recording

#### 2.4.1. Stimuli and procedure

Auditory processing was assessed by means of an electrophysiological non-speech multi-feature paradigm tapping the ability to process rapidly changing and complex auditory stimuli and eliciting responses for two different auditory attributes (i.e., changes in frequency and duration). The paradigm and stimuli were identical to those used in previous research (Cantiani et al., 2016).

Since the paradigm was originally implemented in order to specifically address early Rapid Auditory Processing, pairs of complex tones with a rapid inter-stimulus interval (ISI) of 70 ms were presented. The first tone in the pair always had a fundamental frequency of 100 Hz with 15 harmonics (6 dB roll-off per octave) and 70 ms (5 ms rise time and 5 ms fall time) duration. For standard tone-pairs (STD) the same tone was repeated twice (i.e., 100–100 Hz). Two deviant tone-pairs differing with respect to the second tone were presented: for the frequency deviant (DEVF), the second tone had a fundamental frequency of 300 Hz and 70 ms duration; for the duration deviant (DEVD), the second tone had a duration of 200 ms and a fundamental frequency of 100 Hz.

The stimuli were presented in a passive oddball paradigm where 1,200 stimuli (80% STD, 10% DEVF, 10% DEVD) were pseudo-randomized, so that at least three standard tone-pairs were presented before each deviant pair. The intertrial interval (offset-to-onset, ITI) randomly varied from 700 to 900 ms. All stimuli were presented at an intensity of 75 dB via speakers located on either side of and equidistant (95 cm) from the subject (for a more complete description of the stimuli see Cantiani et al., 2016).

#### 2.4.2. Data acquisition and pre-processing

During EEG recording, children were seated on their caregiver’s lap in a sound-attenuated and electrically-shielded room, watching silent movies or entertained with silent toys. Auditory ERPs were recorded from 60 scalp sites using a dense-array EGI recording system (Electric Geodesic, Inc., Eugene, Oregon) with vertex as the online reference. Sampling rate was 250 Hz with 0.1–100 Hz online bandpass filter.

After recording, EEG data were exported to a MATLAB (Mathworks, Natick, MA) compatible format and processed using EEGLAB (Delorme and Makeig, 2004), and ERPLAB (Lopez-Calderon and Luck, 2014), and custom scripts. An offline bandpass filter of 0.5–30 Hz was used. Noisy channels were interpolated with a spherical spline (never more than 12 of the 60 channels). The signals were then re-referenced to an average reference and the 13 outermost channels were removed due to significant movement-related artifacts and a high rate of interpolation. The remaining 48 channels were considered for analyses. The continuous EEG was segmented according to stimulus type (pre-deviant STD, DEVF, and DEVD) with 100 ms pre-stimulus time (used for baseline correction) and 800 ms post-stimulus time. Bad EEG epochs were identified and rejected using both automatic criteria and visual inspection (for further information of ERP data processing see Cantiani et al., 2016). A minimum of 60 artifact-free trials was used for averaging ERPs (STD condition, *M* = 125.7, *SD* = 23.1, min = 60, max = 184; DEVF condition, *M* = 69.1, *SD* = 9.9, min = 60, max = 104; DEVD condition, *M* = 68.5, *SD* = 10.3, min = 60, max = 102).

#### 2.4.3. Analytic procedure

To examine the role of auditory processing, we focused on the first positive deflection occurring at about 150 ms from stimulus onset (P1) reflecting low-level stimulus detection and registration, and on the following large positive response corresponding to the mismatch response (MMR), reflecting a neural change detection process. Time windows and electrode sites to be submitted to statistical analyses were selected based on mass univariate analyses applied to a subset of ERP data (Cantiani et al., 2016). This procedure allows the identification of channel clusters and time windows where differences between stimulus types are significant, taking into account the application of appropriate corrections for multiple comparisons [for a full description of this procedure and the permutation test results that drove the selection of the time windows and the electrode sites to be submitted to statistical analyses, refer to Cantiani et al. (2016)]. For each participant, ERPs were extracted from a subset of 18 electrodes localized in the left and right frontocentral areas. Data were then averaged in two clusters corresponding to the left and right frontocentral areas, each including nine channels (see Cantiani et al., 2016 for details). Following the above-mentioned mass-univariate analyses, peak (or mean) amplitude was calculated for different time windows:

1. P1: peak amplitude was extracted in the time window 100–300 ms.
2. MMR: we first computed the different waveforms: DEVF-STD (mismatch response for frequency deviant, MMRF) and DEVD-STD (mismatch response for duration deviant, MMRD). Mean amplitude was then extracted in the following time windows: 350–550 ms for MMRF and 420–620 ms for MMRD. Mean amplitude instead of peak amplitude was extracted for the MMR because this component is typically wide and without clearly identifiable peaks.

These measures were available for 132 infants.

### 2.5. Children follow-up

Phonological awareness was measured between 4.5 and 5.5 years of age (*M* = 4.95 years; *SD* = 0.26) using the test “Competenze Metafonologiche” (CMF; Marotta et al., 2008), which is used to evaluate phonological awareness in children from 5 to 11 years old through different tasks. In this study, three subtests were used. (a) Syllabic synthesis: children were asked to pronounce the word resulting from the synthesis of the syllables uttered by the examiner. (b) Rhyme detection: four pictures and their labels were presented to the children; they were then asked to identify the two pictures corresponding to the words that rhymed. (c) Pseudo-word minimal pair discrimination: children listened to recorded pairs of phonologically similar pseudo-words, e.g., “paca”/paka/ and “baca”/baka/, and were asked whether the two stimuli were the same or different. All three subtests refer to and evaluate the same construct, i.e., the ability to work with the sounds of spoken language; moreover, correlations between them were investigated and found to be significant (Syllabic synthesis and Rhyme detection, *r*(101) = 0.281, *p* = 0.001; Rhyme detection and Pseudo-word minimal pairs, *r*(101) = 0.235, *p* = 0.007; Syllabic synthesis and Pseudo-word minimal pairs, *r*(101) = 0.191, *p* = 0.028). Since no specific difference between tasks was expected in relation to the other measures in the study, a composite score made of the mean accuracy score was calculated and entered in the analysis. This measure was available for 103 children.

### 2.6. Statistical analysis

#### 2.6.1. Selection of variables for parental reading skills

The direct assessment of parental reading skills yielded overall six variables (text reading speed; text reading accuracy; word reading speed; word reading accuracy; non-word reading speed; non-word reading accuracy). Descriptive statistics are reported in Supplementary Table S1A. Pearson correlations among variables were computed on the overall sample of parents. Following the correlation matrix (Supplementary Table S1B), we created two composite scores: Reading-accuracy and Reading-speed. These two variables were available from 205 parents (111 mothers and 94 fathers).

Indirect assessment of parental reading skills was performed through the self-report ADCL questionnaire, available on a larger sample (*N* = 344, 172 mothers and 172 fathers). Since the self-report measure yielded significant correlations with both Reading-accuracy [*r*(203) = −0.370, *p* < 0.001] and Reading-speed, [*r*(203) = −0.408, *p* < 0.001], only this self-report measure is reported in the main analysis, in order to maximize sample size.

#### 2.6.2. Preliminary analysis on the ERP data and definition of electrophysiological variables

Descriptive statistics of all electrophysiological variables are reported in Supplementary Table S2A. To identify the electrophysiological variables to be included in the subsequent analysis (in order to try to minimize variables) two preliminary repeated-measures ANOVA models were computed, respectively on peak amplitude of the P1 peak (auditory detection), and on mean amplitude of the MMR (auditory discrimination).

The ANOVA on the P1 peak included Stimulus type (STD vs. DEVF vs. DEVD) and Hemisphere (left vs. right) as within-subject factors. It revealed a main effect of Hemisphere, *F*(1,131) = 38.954, *p* < 0.001, and no effect of Stimulus Type, *F*(2,262) = 0.769, *p* = 0.465 nor the two-way interaction, *F*(2,262) = 1.204, *p* = 0.302. Following these preliminary results, two composite variables were created for the P1 peak, collapsing stimulus types and keeping separate responses in the two hemispheres (P1 Left, P1 Right).

The ANOVA on mean amplitude of the MMR was computed on the difference waveforms, and included Stimulus Type (MMRF vs. MMRD) and Hemisphere (left vs. right) as within-subject factors. It revealed a main effect of Stimulus Type, *F*(1,131) = 15.762, *p* < 0.001, and a significant interaction Stimulus Type x Hemisphere, *F*(1,131) = 4.484, *p* = 0.036. Following these preliminary results, four separate variables were included in the following analysis for the MMR amplitude (MMRF Left, MMRF Right, MMRD Left, MMRD Right).

Despite these differences between hemisphere and stimulus type, all the electrophysiological variables were highly correlated among each other (Supplementary Table S2B).

**Figure 1:**
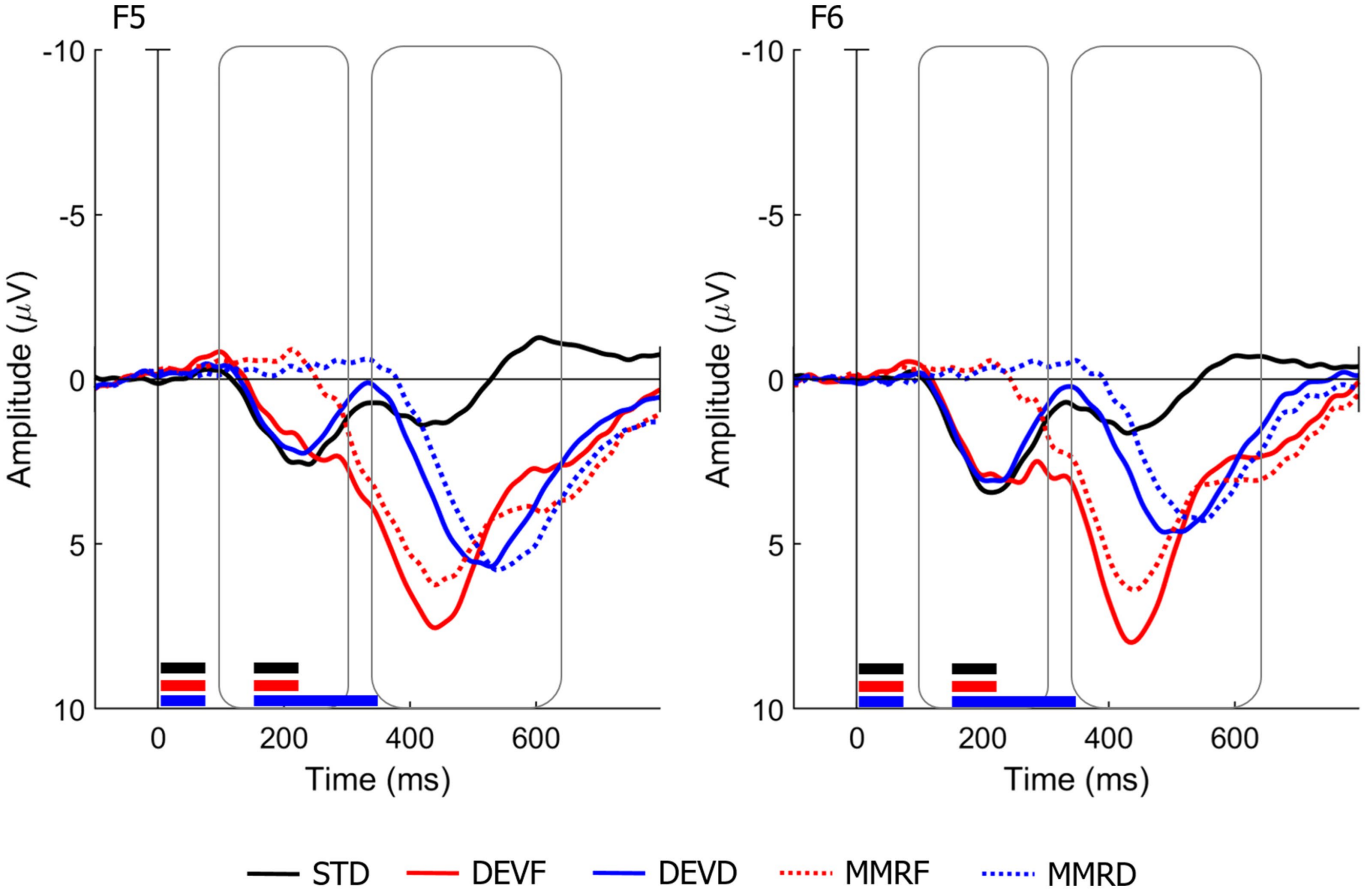
Grand average waveforms for the overall sample. Representative channels for the left and right fronto-central regions are shown. The waveform relative to the standard condition (STD, black line) is plotted against the waveforms relative to the frequency deviant condition (DEVF, red line) and the duration deviant condition (DEVD, blue line). In addition, the difference waveforms relative to MMRF (DEVF minus STD; red dotted line) and MMRD (DEVD minus STD; blue dotted line) are plotted. The black, red and blue lines beneath each waveform indicate the temporal sequence of stimulus presentation in each stimulus type (black lines = STD; red lines = DEVF; blue lines = DEVD). Negative voltage is plotted upward.

Figure 1 shows the grand average waveforms for the overall sample and for two channels (F5 and F6) located, respectively, on left and right frontocentral regions.

#### 2.6.3. Preliminary analysis on socio-demographic variables

Since our second and third experimental questions pertain to the influence of parental reading/musical skills and home environment (specifically literacy and musical environment) on children development, we preliminarily investigated the possible intervening effect of the socio-demographic variables reported and described in Table 1 on the considered parental variables. Based on the literature, we expected higher SES and educational levels to be associated with richer literacy and musical environments (Albert, 2006; Van Steensel, 2006). Spearman correlations were computed among the main socio-demographic variables available (i.e., SES, maternal education, and paternal education) and all parental variables included in the analyses (the full correlation matrix reported in the Supplementary Table S3). As expected, some of our parental variables could be partly explained by socio-demographic variables: home literacy environment was associated with SES and maternal education, maternal reading was associated with maternal education, and paternal reading was associated with both paternal education and familial SES. Conversely, neither parental musical environment nor parental musical skills were associated with SES or educational level. For this reason, and so as to keep the number of variables included in our analyses at minimum, we decided not to include socio-demographic variables in the main analysis.

#### 2.6.4. Analytic procedure

Descriptive statistics and Pearson’s bivariate correlation among the selected variables were performed using SPSS, Version 28.0 (IBM Corp. Released, 2021). These correlational analyses were exploratory and finalized to the generation of more specific data-driven hypotheses to be tested in the following mediation models. Due to the exploratory and preliminary nature of these correlational analyses, we did not adjust significance levels for multiple testing, in order to avoid type-II errors and not to miss potentially relevant associations to be tested in the further models (Bender and Lange, 2001).

Mediation models were tested by using Structural Equation Modeling (SEM) as implemented in the Mplus 8.1 software package (Muthén and Muthén, 2017). We estimated fit indices to determine how adequately the data fit with the chosen models; these indices included the chi-square statistic, the standardized root mean square residual (SRMR, with values ≤0.08 indicating adequate fit), the root mean square error of approximation (RMSEA, with values ≤0.08 indicating adequate fit), and the comparative fit index (CFI, with values of ≥0.95 indicating adequate fit; Preacher and Hayes, 2008). Indirect mediation effects were examined using the bias-corrected 5,000 bootstrap technique to assess non-normality in the product coefficient (Fritz and MacKinnon, 2003). Confidence intervals (95% CIs) that do not contain zero were indicators of significant mediation pathways (Tofighi and MacKinnon, 2011). The mediation models tested two hypotheses. First, we tested the hypothesis that parental skills would influence children’s reading-related outcomes (phonological awareness at 5 years) both directly and indirectly (through early low-level auditory processing skills, as intermediate phenotype). Second, we tested the hypothesis that parental skills would influence the environment provided to children, which in turn would influence children outcomes.

## 3. Results

Descriptive statistics of all the measures included in the analyses are reported in Table 2 (parental measures) and Table 3 (children’s measures).

**Table 2:** Descriptive statistics of parental variables.

	Mothers					Fathers					Combined measures				
	N	Min	Max	Mean	*SD*	N	Min	Max	Mean	*SD*	N	Min	Max	Mean	*SD*
Reading self-report	172	0	9	2.52	2.20	172	0	15	2.90	2.50	344	0	15	2.71	2.36
Home literacy											141	13	62	48.10	7.32
Innovative musical aptitude	87	1	4.14	2.07	0.80	68	1	4.71	2.21	1.01	155	1	4.71	2.13	0.90
Reactive musical behavior	87	1	5	3.77	0.79	68	1.44	4.89	3.22	0.77	155	1	5	3.53	0.83
Musical environment	71	1	660	72.68	94.8	51	0	120	28.16	23.59					

**Table 3:** Descriptive statistics of children variables.

		N	Min	Max	Mean	*SD*
Electrophysiological variables (at 6 months)	P1 Left	132	−0.39	6.43	2.66	1.43
	P1 Right	132	0.28	7.02	3.41	1.58
	MMRF Left	132	−3.36	12.72	3.96	2.94
	MMRF Right	132	−4.01	11.94	3.94	2.93
	MMRD Left	132	−3.26	12.63	3.36	2.76
	MMRD Right	132	−4.01	11.98	2.66	2.84
Phonological awareness (at 5 years)		103	4.00	15.00	10.96	2.40

### 3.1. Are reading skills and musical aptitudes associated?

Associations between reading skills and musical aptitudes were investigated in the overall sample of parents with both measures (*N* = 152, including 85 mothers and 67 fathers with both measures).

Scores in the ADCL questionnaire were negatively associated with both innovative musical aptitude, *r*(150) = −0.223, *p* = 0.006, and with reactive musical behavior, *r*(150) = −0.189, *p* = 0.020. Adults with lower scores in the ADCL questionnaire (corresponding to better self-reported reading skills) were characterized by higher scores in the two subscales of musical aptitude.

### 3.2. Are parental reading skills/musical aptitudes associated with their offspring’s auditory processing and phonological awareness?

Associations between parental measures and offspring electrophysiological measures of auditory processing were measured separately for maternal (*N* = 129) and paternal reading skills (*N* = 129).

As shown in Table 4, a clear pattern of correlations emerged: maternal reading skills were mostly associated with the amplitude of the P1 peak (indexing the detection of the auditory stimuli), whereas paternal reading skills were mostly associated with the amplitude of the MMR, specifically on the right hemisphere (indexing auditory discrimination).

**Table 4:** Pearson’s correlations between parental reading measures and offspring’s auditory processing skills and phonological awareness skills.

		Paternal ADCL (*N* = 129)	Maternal ADCL (*N* = 129)
P1 Left	*r*	0.027	0.207*
	*p*	0.762	**0.019**
P1 Right	*r*	−0.021	0.190*
	*p*	0.812	**0.031**
MMRF Left	*r*	−0.088	0.126
	*p*	0.324	0.154
MMRF Right	*r*	−0.179*	0.030
	*p*	**0.043**	0.736
MMRD Left	*r*	−0.125	0.054
	*p*	0.159	0.544
MMRD Right	*r*	−0.247**	0.027
	*p*	**0.005**	0.759

Significant correlations are reported in bold. Asterisks indicate the level of significance as follows: *p < 0.05; **p < 0.01.

Interestingly, both paternal and maternal reading skills were associated with the offspring’s phonological awareness at 5 years of age, *r*(100) = −0.254; *p* = 0.011, and *r*(101) = −0.248; *p* = 0.013, respectively.

Table 5 shows the pattern of correlations between infants’ auditory processing skills and their later phonological awareness skills. As expected, auditory processing skills at 6 months were significantly associated with phonological awareness at 5 years of age, but only for the amplitude of the MMR, specifically on the right hemisphere.

**Table 5:** Pearson’s correlations between infants’ auditory processing skills and later phonological awareness skills.

		Phonological awareness at 5 years (*N* = 77)
P1 Left	*r*	−0.011
	*p*	0.924
P1 Right	*r*	−0.004
	*p*	0.971
MMRF Left	*r*	0.091
	*p*	0.433
MMRF Right	*r*	0.361**
	*p*	**0.001**
MMRD Left	*r*	0.059
	*p*	0.609
MMRD Right	*r*	0.265*
	*p*	**0.020**

Significant correlations are reported in bold. Asterisks indicate the level of significance as follows: *p < 0.05; **p < 0.01.

Associations between maternal and paternal musical aptitudes, mutually correlated (see Supplementary Table S4A), and the offspring’s auditory processing and phonological awareness skills were assessed in a more modest sample (N ranging between 46 and 58), providing basically no significant correlations with the only exception of a correlation between maternal reactive musical behavior and children’s amplitude of the MMR for the duration in the right hemisphere [*r*(56) = −0.297, *p* = 0.023], and an association between paternal innovative musical aptitude and children’s phonological awareness [*r*(50) = 0.351, *p* = 0.011] (the full correlation matrix is reported in Supplementary Table S4B).

### 3.3. Do parental reading skills and their musical aptitudes influence the environment (respectively the literacy and musical environment) provided to children? How does this environment influence children’s auditory processing and phonological awareness?

Correlations were analyzed separately to explore the relationship between (1) parental reading skills and literacy environment, and (2) parental musical aptitudes (innovative musical aptitude and reactive musical behavior, mutually correlated, see Supplementary Table S4A) and musical environment. Paternal and maternal reading skills were not significantly related to home literacy [*r*(138) = −0.090, *p* = 0.294, and *r*(139) = −0.033, *p* = 0.697, respectively], whereas both maternal and paternal musical aptitudes were related to the musical environment provided by each parent [maternal innovative musical aptitude, *r*(69) = 0.250, *p* = 0.037; maternal reactive musical behavior, *r*(69) = 0.263, *p* = 0.028; paternal innovative musical aptitude, *r*(48) = 0.360, *p* = 0.011], with the exception of paternal reactive musical behavior, *r*(48) = 0.217, *p* = 0.135 that was not related to paternal music environment.

The correlations between literacy and musical home environment and children’s skills show that both literacy and music environment were related to phonological awareness at 5 years of age (home literacy, *r*(92) = 0.230, *p* = 0.027; paternal musical environment, *r*(39) = 0.398, *p* = 0.010, with the exception of maternal musical environment, *r*(51) = −0.120, *p* = 0.390). However, against expectations, the musical environment provided in the first year of life did not influence auditory processing skills at 6 months of age (the full correlation matrix is reported in the Supplementary Table S4B). The influence of home literacy environment on auditory processing skills was not tested, since data collection took place in different time-points (auditory processing at 6 months, and home environment at 3 years).

### 3.4. Mediation models

Based on the correlation matrices, two hypotheses were further explored by means of mediation models.

#### 3.4.1. Auditory processing skills as a mediator between parental reading skills and children’s phonological awareness

**Figure 2:**
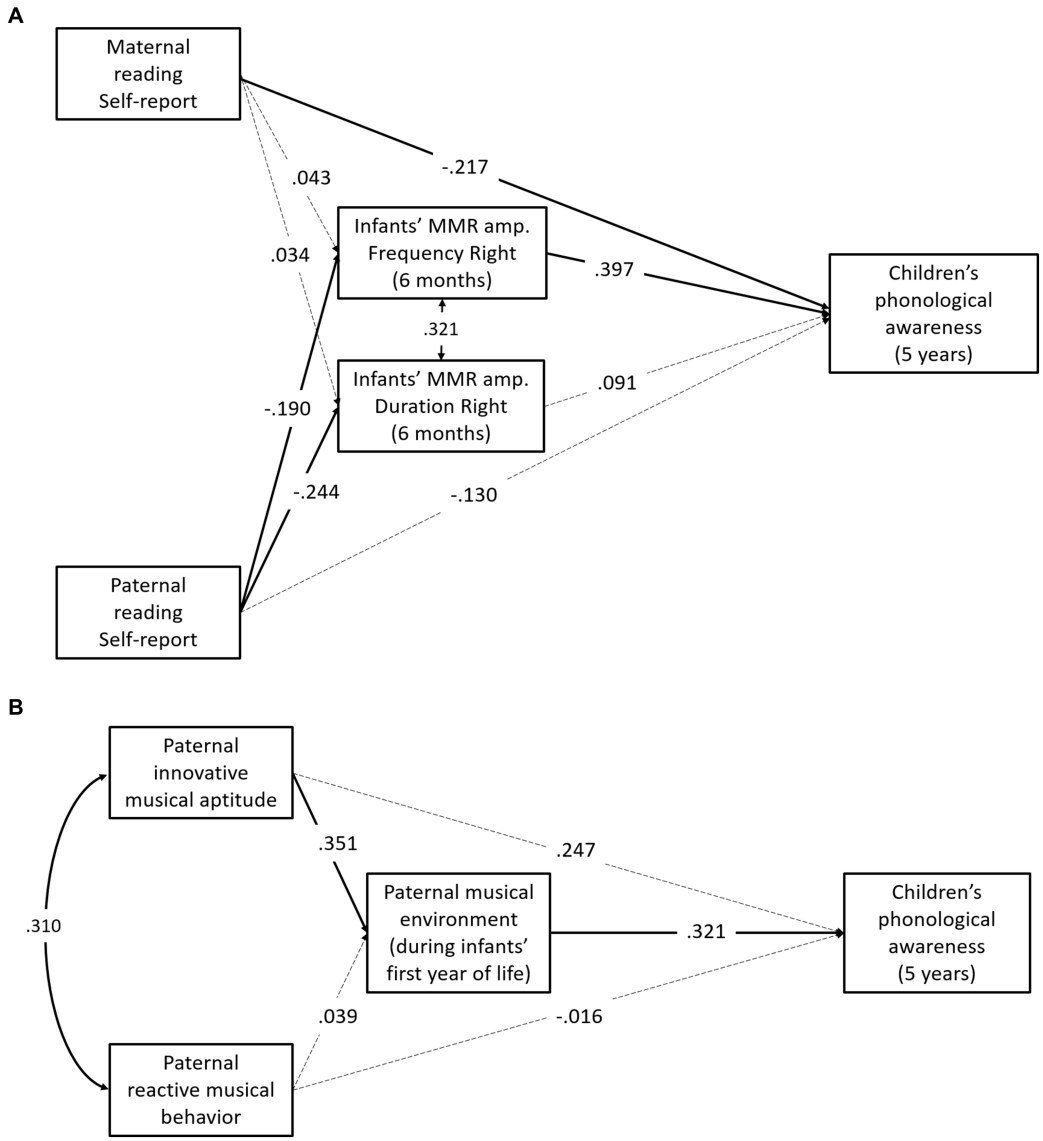
The tested mediation models. Standardized coefficients are reported. Significant paths are presented as continuous lines, whereas non-significant paths are presented as dotted lines.

Having shown that individual differences in children’s phonological awareness were associated with maternal and paternal reading skills and with specific measures of early auditory processing skills in infants (amplitude of the MMR in the right hemisphere), we used SEM to test the mediation model depicted in Figure 2A. The mediation model provided a good fit to the data [X^2^ (9) = 52.752, *p* < 0.001; RMSEA = 0.000, CI (90%) = 0.000–0.000; CFI = 1.00; SRMR = 0.000] and explained 27.9% of the variance in children’s phonological awareness.

Standardized estimates of path coefficients are shown in Figure 2A. The mediation model yielded several significant direct effects. There was a significant path coefficient from amplitude of the MMR in response to frequency stimuli to phonological awareness scores (*β* = 0.397, *p* < 0.001). Children with higher discrimination skills (as reflected in the amplitude of the MMR on the right hemisphere) have higher phonological awareness scores at age 5 years. A significant effect was found from paternal reading skills to both measures of auditory discrimination (MMRF, *β* = −0.190, *p* = 0.017; MMRD, *β* = −0.244, *p* = 0.001): better paternal reading skills predict higher amplitude of the MMR.

More interestingly, using 5,000 bootstrap CI 95%, we found a significant indirect effect from paternal reading to children’s phonological awareness scores via auditory processing skills (*β* = −0.075; SE = 0.037; 95% CI [ −0.148, –0.002]: paternal reading was associated with more positive MMRF at age 6 months which, in turn, influenced phonological awareness skills at age 5 years. Conversely, maternal reading was directly associated with children’s phonological awareness (*β* = −0.217; SE = 0.089; 95% CI [−0.390, –0.043].

#### 3.4.2. Musical environment as a mediator between parental musical aptitudes and children’s phonological awareness

Having shown that individual differences in children’s phonological awareness were associated with parental musical aptitudes and with the musical environment provided by fathers to infants in the first year, we used SEM to test the mediation model depicted in Figure 2B. The mediation model provided a good fit to the data [X^2^ (5) = 18.693, *p* = 0.002; RMSEA = 0.000, CI (90%) = 0.000–0.000; CFI = 1.00; SRMR = 0.000] and explained 21.8% of the variance in children’s phonological awareness.

Standardized estimates of path coefficients are shown in Figure 2B. The mediation model yielded several significant direct effects. There was a significant path coefficient from musical environment to phonological awareness scores (*β* = 0.321, *p* = 0.002). Children whose fathers provided richer musical environment during the first year of life had higher phonological awareness scores at age 5 years. A significant effect was found from paternal musical aptitudes (innovative musical aptitude) to paternal musical environment provided to the children (*β* = 0.351, *p* = 0.003): fathers with higher musical aptitudes provided richer musical environment to their children.

More interestingly, using 5,000 bootstrap CI 95%, we found a significant indirect effect from paternal musical aptitude to children’s phonological awareness scores via the musical environment (*β* = 0.113; SE = 0.058; 95% CI [ 0.022, 0.248].

## 4. Discussion

In the present study, some predictions from the MAPLE framework (Nayak et al., 2022) were investigated in an intergenerational longitudinal design. We attempted to disentangle the complex relations among parental reading skills and musical aptitudes, infants’ neural signatures of auditory processing and later phonological awareness skills, and literacy and musical environment. When interpreting the whole pattern of results, it should be kept in mind that family risk for language or learning impairment was slightly overrepresented in our sample (around 20% of the total sample).

### 4.1. Associations between reading skills and musical aptitudes

The associations between reading skills and musical aptitudes were investigated in the sample of adults included in the study as parents (*N* = 152). Weak but significant associations were found between the two domains (both self-reported). We could have expected the rhythmic properties of musical ability to be specifically related to phonological awareness and reading skills, based on a previous study conducted on developmental population (Politimou et al., 2019). By contrast, the observed associations involved both reactive musical behavior, concerning the sense of rhythm and synchronization, and innovative musical aptitude, concerning the melodic aspects of musical skills. In both cases, self-reported reading skills corresponded to higher musical aptitudes. This result is in line with the large existing literature on the associations between musical abilities and different aspects of typical and atypical language/reading competence across the lifespan (Besson et al., 2007; Bonacina et al., 2018; Boll-Avetisyan et al., 2020; Ladányi et al., 2020). However, the use of self-reported measures and the absence of additional measures on the underlying shared mechanisms are indeed limitations, preventing us from discussing this result further.

### 4.2. Intergenerational associations among parental reading skills/musical aptitudes, infants’ auditory processing, and later children’s phonological awareness

In line with the intergenerational multiple deficit model applied to dyslexia (van Bergen et al., 2014b) arguing that parental cognitive abilities can partly reveal their offspring’s risk, we found that the reading abilities of both parents were predictors of the offspring’s pre-reading (phonological awareness) skills. When looking at the associations between parental reading skills and infants’ neural signature of auditory processing, as a possible endophenotype, an interesting pattern of association emerged. Although, to the best of our knowledge, such a correlational approach has never been applied to similar data, several studies have compared auditory processing skills in infants with and without familial risk for dyslexia, where the risk of dyslexia was quantified based on current parental reading skills (Van Zuijen et al., 2012; Plakas et al., 2013; Thiede et al., 2019). Here, we could add to this literature a potentially different effect of maternal and paternal reading skills.

Maternal reading skills were associated with the amplitude of the P1 peak, the ERP response obligatorily elicited by stimulus occurrence (Čeponiene et al., 2002), reflecting auditory detection. Interestingly, the direction of the association suggested that the amplitude of the ERP peak was higher in infants whose mothers reported worse reading skills, and lower in infants whose mothers reported better reading skills. This finding is novel and deserves further attention in *ad-hoc* studies. Since our paradigm is a long oddball paradigm (overall 1,200 stimuli) characterized by the first stimulus in every pair being always identical, we could speculate that the auditory detection of this first stimulus might be subjected to the phenomenon of repetition suppression, i.e., the decrease of neural activity that can be observed when a certain stimulus is presented repeatedly (Nordt et al., 2016). Here, we can hypothesize that the risk for language and reading difficulties transmitted by mothers reflects itself in a reduced repetition suppression, resulting in an enhanced P1 peak. This explanation is supported by a few studies reporting reduced neural repetition suppression in children with language impairment (Helenius et al., 2014) and with small expressive vocabulary (von Torkildsen et al., 2009). However, further studies specifically manipulating the repetition of the stimuli are needed.

Conversely, paternal reading skills were associated with the amplitude of the MMR, the large positivity peaking at about 300 ms from change detection (Kushnerenko et al., 2002), reflecting the neural change detection process that occurs when there is any detectable auditory change within a sequence of homogeneous sounds. This finding is well-reported in the literature comparing infants with and without familial risk for language and reading impairment (Benasich et al., 2006; Van Leeuwen et al., 2006; Leppänen et al., 2010), where the risk is often estimated based on parental reading skills (Van Zuijen et al., 2012, 2013; Thiede et al., 2019). The same finding of a reduced MMR in at-risk infants was also previously reported using the same or very similar paradigms (Choudhury and Benasich, 2011; Cantiani et al., 2016). Even the hemisphere effect found in the present study (associations with paternal reading are limited to MMR elicited in the right hemisphere) is coherent with what previously found in our Italian sample (Cantiani et al., 2016; Riva et al., 2017). In Cantiani et al. (2016) we compared a sample of 24 6-month-old infants at familial risk for language and learning impairment with a group of 32 infants without such a risk, and found that infants at familial risk were characterized by reduced mean amplitude of the MMR for both deviant stimuli, but only in the right hemisphere. Here, although the approach is different, we found that infants of parents with the worst reading skills behave similarly to infants at familial risk. A speculative explanation for this specific hemisphere effect (that was not found in other studies including similar but not identical paradigms, for example Choudhury and Benasich, 2011) may take into account the specificity of our paradigm, where changes in sound duration produce an irregularity of the rhythm of sound presentation. Such irregularity in the rhythm might require a more substantial activation of the right hemisphere, being significantly more challenging for infants at higher risk. However, this speculative explanation requires further investigation, possibly using analytic strategies that allow age-appropriate source localization (e.g., Cantiani et al., 2019).

The influence of this specific auditory discrimination ability seems to be parent-specific, and driven by fathers. Some forms of parent-of-origin effects, denoting a change in the way an allele may influence a trait dependent on which parent it was inherited from (Lawson et al., 2013) have already been proposed for many complex traits and disorders (Davies et al., 2001; Nudel et al., 2022), including ASD (Flashner et al., 2013; Connolly et al., 2017), and language-related traits and disorders (Nudel et al., 2022). In a recent GWAS on receptive language, a paternal over-transmission of risk alleles was found, with the minor allele considerably reducing language scores when specifically inherited from the father (Nudel et al., 2022).

In our longitudinal work, the pattern of intergenerational associations among maternal and paternal reading skills, infants’ auditory processing at 6 months, and children’s phonological awareness skills at 5 years was further investigated through a more comprehensive structural equation model, revealing that whereas maternal reading skills were directly associated with phonological awareness, the association between paternal reading skills and children’s phonological awareness seemed to be mediated by early auditory processing skills (specifically by the amplitude of the MMR elicited in the right hemisphere in response to frequency deviants). The association from this electrophysiological response at 6 months of age and phonological awareness 4.5 years later was expected based on the original theories of Rapid Auditory Processing (Tallal and Gaab, 2006) predicting the relationship between RAP and language/reading skills through the building of phonological representations and the ability in manipulating them. Our results add to this finding the potential pathogenetic mechanisms in the pathway of familial transmission. However, molecular genetics and twin studies are needed in order to investigate the biological basis of possible maternal and paternal differences.

Associations between maternal and paternal musical aptitudes and the offspring’s auditory and phonological processing skills were investigated in a more modest sample (*N* = 58). Since we hypothesized that music and reading-related traits shared underlying heritable mechanisms, we expected parental musical aptitudes to be related to both infants’ auditory processing and later phonological awareness. Our findings showed that only paternal musical aptitude, concerning the melodic aspects of musical skills, was associated with children’s phonological awareness outcomes. However, infants’ neural signatures of low-level auditory processing did not appear to be influenced by either paternal or maternal musical skills and aptitudes. This result contrasts with previous findings reporting that infants with musically trained parents had better neural entrainment to beat and meter compared to infants with musically untrained parents (Cirelli et al., 2016). On the one hand, the absence of association could reflect a low degree of shared variance between parental musical aptitudes and infants’ auditory processing. It should be noted that the heritability of musical traits has been less investigated and only more recently than the heritability of reading traits. On the other hand, we also should notice that this lack of a relationship cannot be taken as definitive, because of the relatively small sample size for these specific measures.

### 4.3. The interaction between parental reading skills/musical aptitudes and home environment in explaining children’s development

The transmission of parental skills does not only follow genetic pathways but could be passed on also via environmental pathways (van Bergen et al., 2014a). The role of the home environment (specifically the home literacy environment) in children’s language and reading development has been well documented (Burgess et al., 2002; Park, 2008; Dong et al., 2020; Niklas et al., 2020). Here we have explored not only the simple influence of home literacy on children’s development but also the influence of parental characteristics on the environment they are providing to their children. As expected, we found that our measure of the home literacy environment collected at 3 years of age had an effect on children’s phonological awareness around two years later. Surprisingly, however, we found that parental reading skills were not associated with such a measure of home literacy environment. Some previous studies have reported minimal differences between the home literacy environment experienced by young children with and without a dyslexic parent (Elbro et al., 1998; Laakso et al., 1999; Torppa et al., 2007; van Bergen et al., 2014a). Their results showed that experiences of shared reading, print exposure, and cognitive stimulation were similar in families where at least one parent had reading difficulties and in families without such risk. Similarly, in our study, we found that the reading difficulties of the parents did not influence the overall home literacy provided at home (questionnaire filled-up by one of the parents – more often by the mother). These findings suggest that our previously reported association between parental reading skills and children’s phonological awareness is more likely to be explained by a predominantly genetic transmission rather than an environmental pathway. Similar results have been found in previous longitudinal studies, suggesting that genetic transmission and passive gene–environment correlations might be more important than direct environmental effects (van Bergen et al., 2014a). Yet, it should also be considered that, as expected, our preliminary analysis confirmed that both home literacy and parental reading skills correlate with socio-demographic measures such as SES and education level. This suggests that the environmental influence is likely to be more complex than simple exposure to print and music, and may encompass further aspects related to SES and parental educational level, that have not been explored in this study.

The interaction between parental musical aptitudes, musical environment, and children’s development followed a different pattern. Significant associations were found between both parents’ musical aptitudes and the musical environment they each provided to their children in the first year of life: parents who rated themselves as more musically talented were more likely to provide an enriched musical environment for their children. This finding is in line with previous studies reporting that parental musical experience was associated with the habit of listening to music with their child, and with the frequency of playing music and singing to them (Custodero and Johnson-Green, 2003; Ilari, 2005). However, against expectations, the musical environment provided by mothers or fathers did not influence their children’s neural signatures of auditory processing at 6 months of age. To our knowledge, only one study has focused on the relation between informal musical activities carried out at home and children’s electrophysiological indices of neural auditory change detection (Putkinen et al., 2013). This study, which suggests that children whose environment was more musically enriched had developed more mature auditory processing at the neural level, was carried out with older toddlers (2–3 years of age) compared to our participants (6 months of age). In a previous study from our research group, we preliminarily investigated the feasibility and short-term effects of a rhythmic training program proposed to infants (7–9 months) and their caregivers (Dondena et al., 2021). We found beneficial effects on the neural correlates of auditory processing skills. Similarly, Zhao and Kuhl (2016) found enhanced neural responses in infants who participated in similar early musical training. Here, the absence of any effect of the auditory musical environment on the neural responses might suggest that, at least in the first year of life, the type of musical activities [e.g., those specifically related to sensorimotor coupling as in Dondena et al. (2021) and Zhao and Kuhl (2016)] is more relevant than the simple quantity of time spent in any music-related activities.

Concerning the association between early musical environment and children’s phonological awareness skills at 5 years of age, we only found an effect of the musical environment provided by fathers. Although extensive previous research has focused on the beneficial effects of formal music training in preschool or school age on language and reading skills (Moreno et al., 2009; Bhide et al., 2013; Flaugnacco et al., 2015; Cancer and Antonietti, 2022), less is known about the effects of early informal activities that can be carried out by parents at home. Only recently the effect of shared musical activity at home in the first year of life has been reported on language development (Papadimitriou et al., 2021; Franco et al., 2022). For example, Franco et al. (2022) reported that self-reported high levels of parental singing with their infants at 6 months predicted significant advantages on language outcomes (i.e., word comprehension) at 14 months. Similarly, Papadimitriou et al. (2021) reported that both parental singing and overall home musical environment significantly predicted concurrent word comprehension in infants below 12 months, but not in older toddlers. The present result of a long-term effect of informal musical activities carried out by fathers early in development on preschool pre-reading skills, however, is novel. The pattern of intergenerational associations among paternal musical aptitudes, musical environment provided by fathers, and children’s phonological awareness skills was further investigated through a structural equation model, revealing that the association between paternal musical aptitudes and children’s phonological awareness seems to be mediated by the musical environment. Again, this pattern of intergenerational transmission seems to be parent-specific, and more driven by fathers. In addition to the interpretation of a parent-of-origin effect, already proposed for the similar effect found in the paternal reading skills – children’s phonological awareness relationship, a specific effect of the environment provided by fathers emerged for music. A previous study has investigated how fathers’ singing in general is highly engaging to infant listeners (O’Neill et al., 2001), and this might explain the special status of the early paternal influences found in the present study. Here, we found that paternal melodic skills (including singing) have a specific influence on the time they spend in musical activities with their children. Unfortunately, we did not disentangle the specific role of paternal singing, since we collapsed information about listening to music with their child, singing to their child, and lulling their child rhythmically.

### 4.4. Limitations of the study

Although the present study can provide an initial insight on how parental reading and musical abilities both contribute in modulating their offspring’s language development, some limitations should be acknowledged. First, when investigating intergenerational associations using mediation models, different sample sizes were available for parental reading measures (*N* = 129) and musical traits and environment (*N* = 58). Since such differences prevented us from building a solid comprehensive model that could include both aspects, we showed how parental reading and musical traits separately influence children’s phonological awareness through different mediators. Larger and more similar sample sizes are needed in order to investigate how the association between the two traits impacts the intergenerational pathway, i.e., whether it is possible to encompass both parental traits and the environment in a single overall model, and shed more light on how they interact in influencing children’s language competence. A second limitation of the study is the use of self-report measures of parental skills instead of directly assessing them as done with children. Regarding reading skills, however, the ADCL questionnaire is considered as a reliable measure of screening for (suspected) dyslexia in adults (Turner, 2008). Moreover, we directly assessed reading speed and accuracy in a subsample of parents (around ⅔ of the whole sample) using standardized tests, and significant associations between direct and self-report measures emerged. Conversely, we did not test melody and synchronization skills; our estimate of parental musical traits was only based on their self-report answers to the questionnaire. Further studies should also include direct behavioral testing of parental musical abilities. Finally, we should acknowledge that the music environment was only investigated in the first year of life, since we had specific hypotheses on the very early influences that informal musical activities can have on the infants’ brain and language development (Dondena et al., 2021). However, it is well-known that also in preschool years informal home musical experience may have a positive effect on auditory processing (Putkinen et al., 2013), vocabulary (Williams et al., 2015; Schaal et al., 2020) and grammar (Politimou et al., 2019). Recently, a novel tool (i.e., Music@Home) has been developed to assess the home musical environment in the early years comprising an infant and a preschool version (Politimou et al., 2019; Schaal et al., 2020): the longitudinal use of such a tool in further studies may help in disentangling the early vs. later effect of home musical environment.

## 5. Conclusion

To conclude, we present here some findings from an intergenerational longitudinal design and highlight the shared mechanisms underlying parental reading and musical traits, neural underpinnings of infants’ auditory processing, and later phonological awareness skills. Although we overall found associations between reading and musical traits when looking at parents alone, we could only assess the intergenerational transmission effects of these two traits separately, due to the different sample sizes. For reading traits, we confirmed the predictions of an intergenerational transmission to children’s pre-reading (phonological) skills and added some pieces of evidence on the potential mechanisms underlying parent-specific pathways. Specifically, maternal reading skills seem to directly predict children’s phonological awareness, whereas paternal reading skills seem to exert an indirect effect, mediated by infants’ neural underpinning of auditory discrimination skills. Interestingly, the home literacy environment had a significant effect on children’s phonological awareness skills but was not directly associated with either maternal or paternal reading skills. This suggests that the effects of parental reading skills on children’s phonological awareness are more likely to be explained by a predominantly genetic transmission rather than an environmental pathway. As for musical traits, we found again that paternal musical aptitudes and skills, rather than maternal characteristics, were associated with children’s phonological phenotypes: in this case, the association was mediated by the musical environment provided at home. Besides shedding light on possible intergenerational transmission mechanisms concerning reading and musical traits, this study may open up new perspectives for early intervention based on environmental enrichment: our findings suggest that home literacy and musical environment may be suitable targets for early intervention.

## Data availability statement

The raw data supporting the conclusions of this article will be made available by the authors, without undue reservation.

## Ethics statement

The studies involving human participants were reviewed and approved by Ethical Committee of the Scientific Institute IRCCS Eugenio Medea. Written informed consent to participate in this study was provided by the participants’ legal guardian/next of kin.

## Author contributions

CC, MM, and ML designed the study. CC and CD ran the experiment and collected the data and drafted the manuscript. CC, CD, and VR analyzed the data. CC and ML interpreted the results. All authors edited and revised the manuscript.

## Funding

This work was supported by the Italian Ministry of Health (Ricerca Corrente).

## Conflict of interest

The authors declare that the research was conducted in the absence of any commercial or financial relationships that could be construed as a potential conflict of interest.

## Publisher’s note

All claims expressed in this article are solely those of the authors and do not necessarily represent those of their affiliated organizations, or those of the publisher, the editors and the reviewers. Any product that may be evaluated in this article, or claim that may be made by its manufacturer, is not guaranteed or endorsed by the publisher.
